# Promise and Challenge: The Lens Model as a Biomarker for Early Diagnosis of Alzheimer's Disease

**DOI:** 10.1155/2014/826503

**Published:** 2014-02-12

**Authors:** Tian Tian, Boai Zhang, Yanjie Jia, Zhaoming Li

**Affiliations:** ^1^Department of Neurology, the First Affiliated Hospital, Zhengzhou University, Zhengzhou 450052, China; ^2^Department of Oncology, the First Affiliated Hospital, Zhengzhou University, Zhengzhou 450052, China

## Abstract

Alzheimer's disease (AD) is the most common form of dementia pathologically characterized by cerebral amyloid-beta (A*β*) deposition. Early and accurate diagnosis of the disease still remains a big challenge. There is evidence that A*β* aggregation starts to occur years before symptoms arise. Noninvasive monitoring of A*β* plaques is critical for both the early diagnosis and prognosis of AD. Presently, there is a major effort on looking for a reasonably priced technology capable of diagnosing AD by detecting the presence of A*β*. Studies suggest that AD is systemic rather than brain-limited focus diseases and the aggregation of the disease-causing proteins also takes place in lens except the brain. There is a possible relationship between AD and a specific subtype of age-related cataract (supranuclear cataract). If similar abnormal protein deposits are present in the lens, it would facilitate non-invasive diagnosis and monitoring of disease progression. However, there are controversies on the issues related to performance and validation of A*β* deposition in lens as biomarkers for early detection of AD. Here we review the recent findings concerning A*β* deposition in the lenses of AD patients and evaluate if the ocular lens can provide a biomarker for AD.

## 1. Introduction

Alzheimer's disease (AD) is a progressive and fatal neurodegenerative disorder and the leading cause of dementia worldwide [1]. The diagnosis of definite AD is based on the presence of characteristic features including beta-amyloid (A*β*) plaques performed postmortem [2–4]. A*β* peptide, generated by endoproteolytic cleavage of the *β*-amyloid precursor protein (*β*-APP), is composed of 39–43 amin acid residues long [5]. Studies suggest that the rate of A*β* deposition is associated with significant memory decline and disease progression in patients with AD [6]. A*β* deposition starts to occur years before cerebral atrophy and cognitive decline [6–8], so this potentially affords a wide time window for intervention with anti-A*β* therapy. However, early and accurate diagnosis of AD still remains a big challenge and the lack of reliable method for its early diagnosis hampers the efficient drug development seriously.

Presently, there is a major effort investigating the promising novel approaches for AD early diagnosis by detecting the presence and accumulation of protein A*β*. Brain scans such as magnetic resonance (MR) microscopy [9], positron emission tomography (PET) tracers [10–12], and spinal fluid tests [13] are currently used by researchers to evaluate Alzheimer's-related changes and monitor disease course in living AD patients and animal models, but they are costly and impractical for widespread screening, and none of them have been approved for routine use [14, 15]. There is a clear need to look for a reasonably priced technology capable of detecting the condition before symptoms occur with high resolution sensitivity and specificity that offers ease of use during the test.

AD has been identified as one of the conformational diseases whose etiology involves aggregation of misfolded protein in brain, for example, deposition of A*β* peptides in the brain of AD [16]. AD is a systemic rather than brain-limited focus disease [17]. In addition to the brain, the aggregation of A*β* was also detected in ocular structures, especially lens [18–20]. The conformational alteration in lens proteins may induce cataract formation [21, 22]. The slightest opacity in any region of the lens can be a sign of systemic disorders [23]. Studies suggest a possible relationship between AD and a specific subtype of age-related cataract (supranuclear cataract) [19] and the existence of a pathway leading to AD-linked pathology in the brain and lens [24]. What is more, the frequency of dementia in patients who had cataract is higher than that in patients without cataract [25]. It is therefore speculated that cataract can be considered a window to indicate the presence of systemic disorders. Currently, ocular lens model as a biomarker for applicable diagnostic testing are under intensive investigation. However, there are uncertainties and controversies on the issues related to performance and validation of A*β* deposition in the lens as a biomarker for early detection of AD. Here we review recent researches on the A*β* deposition in lenses of AD patients and evaluate if the ocular lens can provide a biomarker for patients suffering from AD.

## 2. Advantages of the Lens as a Biomarker for AD

Eye is the “window” into the brain. Given the direct connection of ocular tissues to the brain, the eye would be an attractive site for early diagnosis and monitoring of neurodegenerative diseases. The lens is a unique immune-privileged tissue containing transparent cells and is considered to be an ideal environment for protein aggregation [26]. The human lens is susceptible to age-related degenerative changes and shows progressive deposition of insoluble protein, which induces the formation of cataract [27]. There are a variety of highly sensitive noninvasive optical techniques for the eyes, including slit lamp examination, and optical coherent tomography. The cataract can be easily detected and tracked by noninvasive and highly sensitive optical techniques, so the lens would provide an ideal “window” to monitor the molecular progression of AD. The slightest opacity in any region of the lens may be a sign of systemic disorders.

To determine if this is Alzheimer's cataract, researchers can inject special fluorescence drops that bind A*β* proteins into the eye [1]. Importantly, both lens and brain originate from epithelial tissues (surface ectoderm and neuroectoderm, resp.), and the ocular lens retains a stem cell population that proliferates and differentiates throughout life. It does not destroy old cells by apoptosis but uses young stem cells to continually nurture older cells. With the long-lived nature of the terminal differentiated cell types, the lens provides a record of the life history of the individual from embryo to old age [24]. Longevity of expressed A*β* in the lens fiber cells, the inefficient protein turnover capacity of mature lens fiber cells, and optical accessibility of the lens suggest that the cataract induced by A*β* aggregation is detectable throughout the course of AD. Such tests will advance the development of new therapies for AD and maximize the therapeutic potential of emerging drugs.

## 3. Does A*β* Accumulate in the Lenses of AD?

Current studies have been focused on detecting A*β* in the lenses to aid in the diagnosis of AD; however, the results are different (Table 1).

Alzheimer pathophysiology has been noted to contribute to cataract formation [28]. Goldstein et al.'s study was the first to discover evidence of AD-associated pathology outside the brain. They demonstrated the regionally-specific A*β* deposits in the supranuclear region of the ocular lenses of patients with AD in whom A*β* had been confirmed by autopsy, but not in those without the disorder [19]. Supranuclear cataract is a particular uncommon phenotype of cataract just above the center (the nucleus) of the lens. Most age-related cataracts manifest as nuclear sclerosis or cortical cataracts. Nuclear sclerosis is the most common type of cataract that involves the central or “nuclear” part of the lens. Cortical cataracts are caused by opacification of the lens cortex (outer layer) [29]. Supranuclear cataract is different from those commonly age-related cataracts and appears to be specific for AD. A*β* hetero-oligomeric aggregate was detected within the cytoplasm of equatorial supranuclear lens fiber cells [19]. Similar results have also been found in Down's syndrome (DS) (trisomy 21) subjects with early onset AD [30]. The DS phenotype complex is a condition with features of premature aging, a high frequency of AD, and distinctive early-onset cerulean cataracts of unknown etiology [31, 32]. The study revealed a characteristic pattern of supranuclear opacification in DS lenses accompanied by accelerated supranuclear A*β* deposition and fiber cell cytoplasmic A*β* aggregates identical to the lens pathology identified in AD [30]. These findings confirmed that the classical amyloid pathology that goes on in the brain also occurs in the lens. A*β* plaque in the lenses is the cause of cataracts in these patients. Moreover, increased A*β* accumulation is a key pathogenic determinant linking lens and brain pathology in both AD and DS [19, 30]. Furthermore, the concentrations of A*β* in the human lens and aqueous humor are comparable with those in aged human cerebral cortex and cerebrospinal fluid, respectively [19, 33]. In addition to the establishment of A*β* colocalization with a specific type of cataract in AD patients, as mentioned earlier [19], transgenic mouse models of AD developed more cataract with enhanced A*β* immunoreactivity than wild-type mice [18, 20]. Besides, A*β* deposition has also been detected in the lenses of rats, monkeys, and mouse models of Down's syndrome (human amyloid-*β* protein precursor transgenic mice) [28, 34]. Taken together, these discoveries suggest possible overlapping AD-associated A*β* pathology in the lens and brain. AD is a more global, systemic disease rather than one confined to the brain. Based on these data, as well as the ectodermal embryological origin of both the lens and brain, and the long-lived nature of the terminal differentiated cell types affected by AD pathology in the lens [24], there is a possibility that an ocular biomarker for AD may exist in lens and raises hopes of simple ophthalmic testing.

Recently, Kerbage et al. evaluated that if the SAPPHIRE System (a combination of a fluorescent ligand and a laser scanning device) can aid diagnosis of probable AD by detecting the presence of A*β* in vivo of a human lens. In the study, the ointment was placed in the eye and it bound to A*β* deposits to produce a fluorescent signal visible by laser scans. The results showed that the specific fluorescent signature of ligand-marked A*β* in the supranuclear regions was higher among AD patients as compared to normal controls, and no serious adverse events were observed. The average signal detected in AD patients was approximately twice that of the control, and the deeper supranuclear area revealed higher signal as compared to other regions in the supranuclear [1]. The results are encouraging in terms of bioavailability of the ligand in the anterior segment of the eye and the sensitivity of the device to distinguish AD patients from healthy controls. What is more, it complements the previous study by Goldstein et al. to detect A*β* plaque in the supranuclear region of lenses from AD subjects.

While clinical trials are being carried out, other researchers failed to replicate the original publication. A recent study by Michael and colleagues who examined lens tissue of 21 donors with AD (17 clinically diagnosed AD patients, four neuropathologically confirmed AD patients) was unable to find any sign of A*β* in the lens samples using immunohistochemical and amyloid stains [35]. The same results were found in another independent research conducted by Ho et al. They reported that no amyloid deposit was identified in the lenses or other structures in the eyes of AD patients, while plaques with strong A*β* staining were present in the brains of these affected subjects [36]. These new data cast doubt on the usefulness of the lens as an early indicator for AD and raise issues about the robustness and reliability of it for widespread use.

The reasons for these contradictory findings are not entirely clear. By comparing these studies, we find that there seem to be major differences in the quality of the stained images between them, suggesting that the reasons lie partly in the staining procedures (Bennhold's technique used by Goldstein et al. versus Puchtler's technique used by Michael et al., who also used a different antibody for immunostaining). Besides, the differences in postmortem interval, types of cataracts, primary fixation of the lenses, frozen tissues, and whole mount preparations may also account for some of these discrepancies. Furthermore, cataract, as another conformational disease, is also affected by many other complicated factors and the mechanism of A*β* plaque formation in the lens is unknown. What is more important, it's not known if A*β* deposits in the lens precede the symptoms of AD. To sum up, it is still uncertain and controversial if A*β*  deposition in the lens can provide a biomarker for AD.

## 4. Perspectives of Early AD Diagnosis and Treatment

At present, definitive antemortem AD diagnosis is difficult and there is still no cure for the disease. Evidence indicates that neuropathological abnormalities associated with AD, especially A*β* accumulation, may occur during the prodromal phase, as much as decades before the appearance of the first cognitive symptoms [37]. Developing a reliable, practical, and cost-effective diagnostic methodology for AD would facilitate its early diagnosis, accelerate preclinical drug discovery, and therefore allow early effective intervention with new drugs, so as to delay disease onset, prevent neurodegeneration, or modify the course of AD. In this respect, the unequivocal identification of A*β* specific plaques is formed in AD patients' lenses and share properties with those in the brain, especially at an early stage, and the noninvasive in vivo detection of individual plaques deposition in lenses from AD patients could develop specific biological biomarkers for early AD diagnosis, prognosis assessment, and response to therapies. In addition, understanding the impact the AD process has on the visual system can result in more effective communication with members of the AD multidisciplinary team.

## 5. Implications for Future Research

Even though MR imaging and PET tracers have become common in many hospitals, it is never easy to evaluate A*β* protein aggregation in CNS in a noninvasive manner. If AD-linked lens pathology actually reflects the changes in the brain, monitoring the neurodegeneration in the brain can be a simple task. Although great progress has been made in this area, there still remain significant questions. Firstly, A*β* plaques have only been detected in the lenses of human AD subjects in three independent studies [1, 19, 30], so the findings need to be further corroborated. Secondly, there is no evidence suggesting that lens pathology precedes brain pathology. In the future, large-scale well-designed trials include a larger number of participants that are needed to determine the specific mechanisms underpinning presumptive linkage of AD pathology in these two anatomical compartments and to test lens A*β* as a probable diagnostic tool for AD. Also, the development of more sensitive technology will affiliate the detection of very earliest stages of A*β* in lens. In addition, animal models have contributed and will continue to contribute to the key understanding of neurodegenerative diseases, despite anatomical and physiological differences [18, 20, 34, 38]. Studying the relationship between ocular structures and AD is an easier task in animal models than in humans. The time and cost are significantly reduced when compared to studying the relation in humans and hence will increase the chance that a correct link will be established between cataract and AD. Determining the correct mechanism and link between them will introduce a whole new direction of research into treatment, intervention, and early detection of AD or cataract.

## 6. Conclusions

At present, early and accurate presymptomatic diagnosis of AD remains a big challenge. It is suggested that the lens would be a novel, accessible AD biomarker to study disease-relevant proteins accumulation with optical test and therefore can provide a new quantitative AD diagnostic technology. Studies related to performance and verification of A*β* deposition in lens for applicable diagnostic testing are under intensive investigation. However, the results are different from each other. It is still uncertain if the ocular lens can provide a biomarker for early detection of AD. The potentially crucial technical differences may underlie the lack of replication in these studies.

A sensitive noninvasive test for AD will accelerate preclinical drug discovery and facilitate diagnosis and progression monitoring. Patient care will be greatly enhanced by early diagnosis, initiating early therapy, monitoring disease process and assessment of therapeutic effect. The lens model is still promising, but more data on prospective human validation trials in larger samples are needed to reveal if changes in the lens are preceding the symptoms of AD and if the SAPPHIRE System can serve as a useful diagnostic tool to detect AD at an early stage.

## Figures and Tables

**Table 1:** Studies on detecting A*β* in the lenses of patients with AD.

Title	Year & authors	Patients	Significance
Cytosolic *β*-amyloid deposition and supranuclear cataracts in lenses from people with Alzheimer's disease	2003, Goldstein et al. [19]	AD patients	AD patients suffered from a specific type (supranuclear) of cataract; A*β* was present in the cytosol of supranucleus lens fibre cells of those people with AD.

Alzheimer's disease amyloid-*β* links lens and brain pathology in Down's syndrome	2010, Moncaster et al. [30]	Down syndrome (DS) subjects with early onset AD	Increased accumulation of A*β* in lenses caused cataract in DS; increased A*β* accumulation is a key pathogenic determinant linking lens and brain pathology in both DS and AD.

Absence of beta-amyloid in cortical cataracts of donors with and without Alzheimer's disease	2013, Michael et al. [35]	AD patients	No A*β* was detected in lens samples from donors with AD.

Beta-amyloid, phospho-tau, and alpha-synuclein deposits similar to those in the brain are not identified in the eyes of Alzheimer's and Parkinson's disease patients	2013, Ho et al. [36]	AD patients	No A*β* was detected in the lenses of AD patients. A*β* does not aggregate in the eye in a manner analogous to the brain or is present at lower levels or in different forms.

Alzheimer's disease diagnosis by detecting exogenous fluorescent signal of ligand bound to beta amyloid in the lens of human eye: an exploratory study	2013, Kerbage et al. [1]	AD patients	The specific fluorescent signature bound A*β* in the supranuclear areas was higher among AD patients as compared to controls; the deeper supranuclear region revealed higher signal as compared to other supranuclear regions; the SAPPHIRE System can aid diagnosis of probable AD by detecting the presence of A*β* in vivo of human lens.
